# Both lipopolysaccharide and vehicle treatments upregulate complement-dependent and complement-independent innate immune responses in Diamond-backed Watersnakes (Squamata: *Nerodia*)

**DOI:** 10.3389/fimmu.2026.1820243

**Published:** 2026-04-01

**Authors:** Jennifer Terry, Emily K. Field Rezac, Grant Dawson, Katherine Willis, Kevin J. Krajcir, Stephen J. Mullin, Lorin A. Neuman-Lee

**Affiliations:** 1 Department of Biological Sciences, Arkansas State University, Jonesboro, AR, United States; 2 Mississippi Department of Wildlife, Fisheries, and Parks, Mississippi Museum of Natural Science, Jackson, MS, United States; 3 Department of Microbiology and Immunology, University of Arkansas for Medical Sciences, Little Rock, AR, United States; 4 Stiles-Nicholson Brain Institute, Florida Atlantic University, Jupiter, FL, United States; 5 Arkansas Natural Heritage Commission, Department of Parks, Heritage, and Tourism, Little Rock, AR, United States

**Keywords:** antimicrobial peptides, innate immunity, leukocyte, LPS, reptile

## Abstract

**Introduction:**

The innate immune response is complex and highly context dependent. In reptiles, relatively little information is available regarding how individual innate immune components interact and change over the course of an immune response.

**Methods:**

We characterized innate immune responses of Diamond-backed Watersnakes (*Nerodia rhombifer*) over a 72-hour period after stimulation by a nonpathogenic antigen, lipopolysaccharide (LPS). Blood samples taken at predetermined time points were used to determine microbial killing ability of immune cells and proteins. Using two microbes that elicit unique responses (gram-negative *Escherichia coli* and gram-positive *Staphylococcus aureus*), we explored the contribution of unique immune components through a series of microbial killing assays using fresh versus frozen-thawed serum and buffy layer (serum+BL).

**Results:**

There was an effect of handling on the immune activity of the fresh serum+BL (leukocyte-dependent responses and proteins) as snakes across treatments showed increased response to *E. coli* and a decreased response to *S. aureus*. LPS-treated snakes had a stronger immune response overall to both *E. coli* and *S. aureus* when only proteins were measured.

**Discussion:**

Our findings demonstrated the importance of including both vehicle saline injection and handling-only control groups in manipulative studies as the patterns for these two groups differed. This study describes the response of multiple metrics of innate immunity to different immune challenges over a 72-hour period, which provides novel insight into the immune response of a poorly understood taxon. Overall, this study provides evidence that non-antigenic saline vehicle injections stimulate an immune response and that leukocyte-dependent and complement responses are time-specific following an immune challenge.

## Introduction

The innate immune response is complex and multifaceted. Immune responses and specific components of the immune system in vertebrates are highly context-dependent and influenced by various life-history and environmental factors (1, 2). Yet, in order to quantify the influences on the immune system, the basic functions of the immune system must be understood. Reptilian immune responses have received less attention than other taxa (3, 4), which limits our understanding of variation in these responses under different contexts. In reptiles, innate immune responses are generally robust whereas adaptive responses are moderate (3, 5). The dynamics of the reptilian immune response are not well understood, however, because members of this clade are not typically considered model organisms in physiological studies (6). We lack the fundamental knowledge of how different innate immune components interact and mobilize in response to infection in reptiles.

All free-living organisms have limited access to energetic resources and, as such, must efficiently allocate these resources among fundamental processes such as immunity. Although the ability to efficiently fight off an infection is critical to survival (7), an individual cannot invest maximally in all immune defenses. Instead, certain immune components can be mobilized over others depending on the type of infection or context (8–11). Several key components of reptilian innate immunity are mobilized preferentially: the complement system, leukocyte-dependent responses, natural antibodies (NAbs), and antimicrobial peptides (AMPs). The complement system comprises a cascade of proteins present in the serum that help destroy invading bacteria via opsonization, lysis, and/or inducing inflammation (12). Prior work suggests that complement responses are critical in combatting bacterial infections in reptiles (13–15). Leukocyte-dependent responses in reptiles involve a suite of non-specific leukocytes such as heterophils (similar to neutrophils in mammals), lymphocytes, and azurophils (a unique leukocyte most prominently found in snakes (16, 17). NAbs are an alternative antibody response found in individuals with no known prior antigenic exposure (3, 18). AMPs are a diverse class of peptides with a wide range of inhibitory mechanisms against bacteria, fungi, parasites, and viruses (19, 20). While these components have been studied to some extent in reptiles (e.g., (4, 21), the interactions between components during an immune response have not been well assessed.

**Figure 1:**
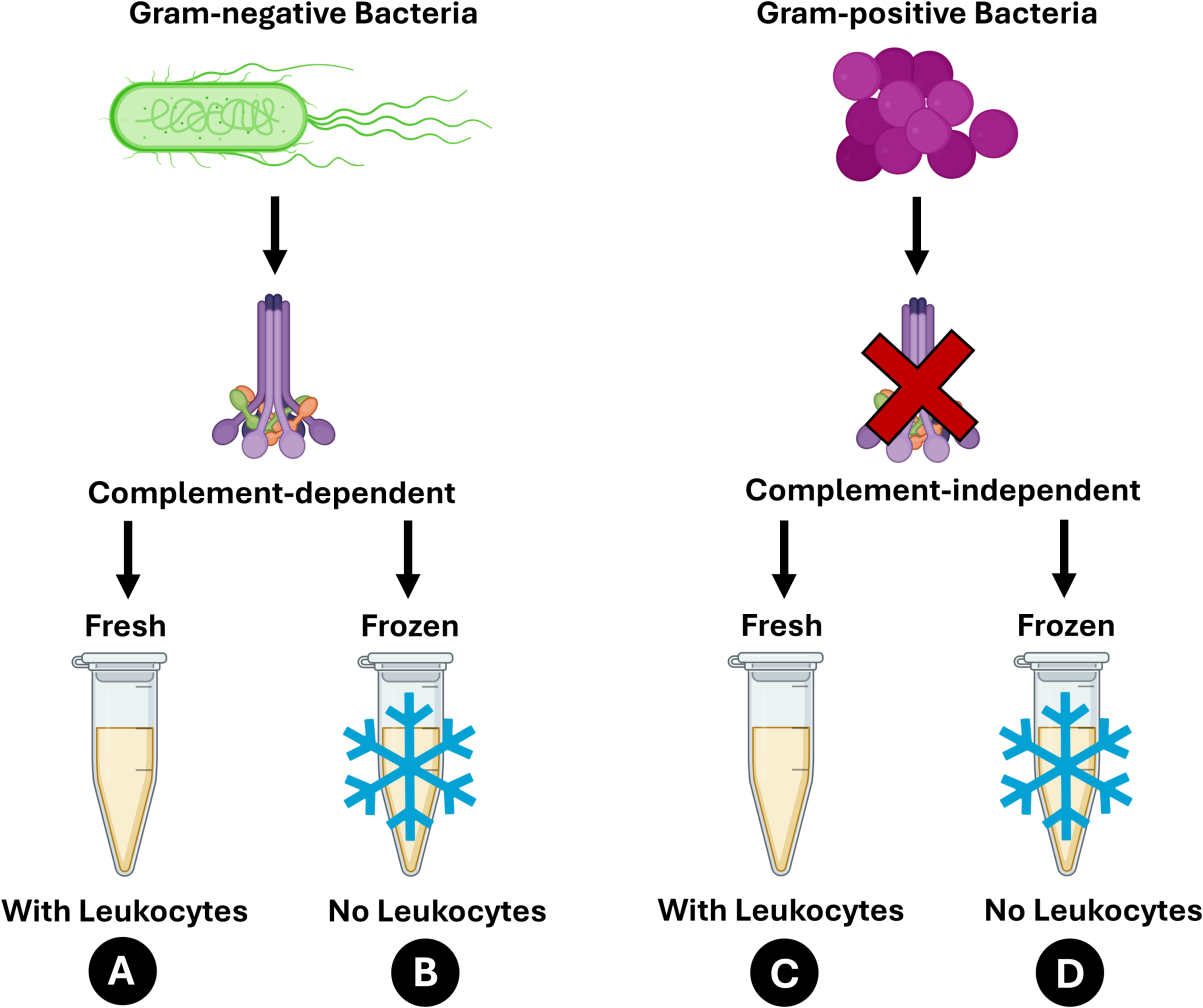
Microbial killing ability assay manipulations by using gram-negative (*Escherichia coli*) versus gram-positive (*Staphylococcus aureus*) bacteria and serum+buffy layer manipulation (serum+BL; fresh versus frozen-thawed) to assess the contribution of leukocyte-dependent responses, the complement system, antimicrobial peptides (AMPs), and natural antibodies (NAbs). **(A)** Fresh serum+BL inoculated with gram-negative bacteria assesses the contribution of all immune components. **(B)** Frozen serum+BL inoculated with gram-negative bacteria removes the contribution of leukocyte-dependent responses and assesses the contributions of the complement system, AMPs, and NAbs. **(C)** Fresh serum+BL inoculated with gram-positive bacteria removes the contribution of the complement system and assesses the contributions of leukocyte-dependent responses, AMPs, and NAbs. **(D)** Frozen serum+BL inoculated with gram-positive bacteria removes the contributions of leukocyte-dependent responses and the complement system and assesses the contributions of AMPs and NAbs. Graphics from BioRender.

The overall innate immune response is the cumulative input of each innate immune component. Microbial killing assays (MKAs) assess the overall immune response by measuring the relative change in the amount of microbe (either grown or killed) after adding a known volume of sample (e.g., blood serum) (22). Any microbe killed by a fresh sample is a result of the total contributions of leukocytes, the complement cascade, AMPs, and NAbs (23). The extent to which each of these components contributes to the immune response can be elucidated through sample manipulation and microbe selection in MKAs (**Figure 1**). The contribution of the complement system can be determined by comparing the clearance of gram-negative and gram-positive bacteria. For example, *Staphylococcus aureus*, a gram-positive bacterium, has evolved various strategies of escaping detection by the complement system (24–26). Thus, the use of *S. aureus* in the MKA is an effective technique in assessing non-complement innate immune responses. *Escherichia coli*, a gram-negative bacterium, is the most common microbe used in the MKA and assesses complement-dependent immune responses because this microbe is less effective at evading complement responses (10, 27, 28). The contribution of leukocytes can be measured by comparing the microbial killing abilities of fresh versus frozen-thawed sample because frozen-thawed samples will not have leukocyte-dependent responses (2, 29). The relative contribution of the complement cascade, leukocyte-dependent responses, AMPs, and NAbs can be assessed by systematically removing innate immune components through these assay manipulations (23).

Experimental approaches to immune studies can provide insight into the relative contribution of an organism’s immune components over the course of an immunological response. For example, gram-negative bacteria are recognized by the pathogen-associated membrane pattern (PAMP) lipopolysaccharide (LPS) (30, 31). Isolated LPS can be used as a non-pathogenic antigen to simulate a bacterial infection and activate an immune response (32–34). Using a non-pathogenic PAMP such as LPS can allow researchers to examine an immune response in a more controlled manner, which helps establish basic immunological patterns across contexts and in poorly understood taxa (15).

In the present study, we examined the innate immune response in male Diamond-backed Watersnakes (*Nerodia rhombifer*) to a simulated infection using LPS, a non-pathogenic antigen. We assessed the immune response over 72-hours and performed a series of manipulative MKAs targeted at systematically removing contributions of individual immune components by using fresh versus frozen-thawed serum and buffy layer (serum+BL) and gram-negative (*E. coli*) versus gram-positive bacteria (*S. aureus*). We predicted that (1) LPS-treated snakes would exhibit overall stronger immune responses, (2) patterns of immune response would differ between complement-dependent and complement-independent responses, and (3) immune responses would change over the course of the simulated infection.

## Methods

### Animal collection

Twenty-nine male *N. rhombifer* were hand-captured from 11–15 April 2022, from Coldstream Fisheries Farm in Greene County, Arkansas, USA, and transported in individual opaque bags to Arkansas State University within 4 hours of capture. This species is appropriate for addressing our questions because it is broadly distributed throughout the southeastern U.S., occurs in a variety of freshwater habitats (with considerable variation in water quality), and is representative of a speciose clade of nonvenomous snakes (35). Upon arrival in the laboratory, we measured the mass (± 0.1 g) of each individual and bathed each snake in diluted betadine. We housed snakes separately in molded plastic tubs with newspaper substrate and a water dish in a reptile rack storage system. Air temperatures were maintained at 21.1°C with a heat tape gradient set from 21.1–29.4 °C and the photoperiod was set to a 12:12 hour light:dark schedule. Snakes were offered one meal of a single feeder goldfish (*Carassius* sp.) within a week of arriving in the laboratory. All uneaten food was removed after 24 hours. Body condition was assessed via visual inspection for all individuals before the start of the experiment to ensure all animals were in similar condition (e.g., not emaciated or significantly overweight). All procedures were approved by the Arkansas State University IACUC (Protocol # FY21-22-34). Prior to subject collection, an Arkansas scientific collection permit for reptiles and amphibians was obtained from the Arkansas Game and Fish Commission (permit # 022320222).

### Experimental design, agents, and dosages

All subjects were housed for a maximum of two weeks before their involvement in the experiment. Prior to treatment, each snake was weighed, and a baseline blood sample (1 mL) was collected via the caudal vein within three minutes of removal from the enclosure (36, 37). Snakes were randomly assigned to control (n = 9), vehicle solution (n = 10), and LPS (n = 10) treatment groups. In the randomization, size was evenly distributed prior to finalizing treatment groups so that mean subject mass was similar in all treatments. Purified LPS (lyophilized powder prepared by phenol extraction) from the cell wall of the bacteria, *E. coli* (serotype O127:B8; Sigma Aldrich, MA, USA) was dissolved in phosphate buffered saline (PBS). Treatments were administered immediately following baseline blood sample collection. Injection volume (1 ul solution/1 g subject mass) was calculated for each individual as 2.5 ug/g of subject mass on its capture date (38), and solutions were placed into the subject’s coelom posterior to the stomach using a 26.5-gauge needle. Injection volumes for vehicle treatments (PBS only; hereafter referred as “saline”) were calculated in the same manner (1 ul solution/1 g subject mass). Control individuals were removed from their enclosures and probed with a capped syringe to simulate injection. Snakes were returned to their housing bins following treatment.

Further blood samples (1 mL) were collected at 12-, 24-, 48-, and 72-hours post-treatment. All blood samples were collected within three minutes of subject removal from its respective bin. Samples were aliquoted into a non-heparinized 2.0-mL microcentrifuge tube. Blood was centrifuged for 10 minutes at 10,000 rpm and the serum+BL was removed from the red blood cells via pipette. Serum+BL was aliquoted into two 0.6-mL microcentrifuge tubes; one tube was maintained for < four days at 4°C for fresh serum+BL MKAs and one tube was frozen for approximately one month at -20°C for frozen serum+BL MKAs. All frozen-thawed samples were subjected to one freeze-thaw cycle.

### Microbial killing ability assays

We performed microbial killing assays (MKAs) in triplicate using a modified protocol optimized for snakes to assess innate immunocompetence (10, 39). Non-complement immune responses were assessed using *S. aureus*, whereas complement-dependent immune responses were measured using *E. coli* (28). Leukocyte-dependent responses were assessed by measuring fresh serum+BL. Ultimately, we assessed the relative contribution of leukocyte-dependent responses, the complement system, AMPs, and NAbs by systematically removing each component (23) (**Figure 1**). To assess the overall immune response with contribution from all immune components, we performed MKAs using fresh serum+BL and *E. coli*. We also performed MKAs using frozen-thawed serum+buffy layer and *E. coli* to remove the contribution of leukocyte-dependent responses. To remove the contribution of the complement system, we performed MKAs using fresh serum+BL and *S. aureus*. Lastly, to remove both leukocyte-dependent responses and the complement system, we performed MKAs using frozen-thawed serum+BL and *S. aureus*, which left only the contributions of AMPs and NAbs.

Briefly, to conduct our MKAs, we combined 6 μl of serum+BL with 12 μl 10X PBS and 6 μl 1 x 10^5^ colony-producing units *E. coli* or 1 x 10^5^ colony-producing units *S. aureus* on a sterile 96-well microplate. We incubated plates for 30 minutes at 37 °C and added 125 μl 3% tryptic soy broth to each well. We used a microplate absorbance reader (340 nm, Biotek Instruments, Inc., VT, USA) to calculate baseline absorbance. Plates were then incubated for an additional 12 hours before being read again to measure post-incubation absorbance. Percent MKA (%MKA) was calculated by dividing the mean change in absorbance (i.e., post-incubation minus baseline) for each sample by the mean change in absorbance for the positive controls (i.e., bacterial solution and media only). All values below 0 or above 100 percent killing were adjusted to 0 and 100 respectively. Negative controls (media only) were run on each plate to ensure contamination was absent.

### Statistical analyses

We conducted all statistical tests in Program R (40) and interpreted results at a significance of α = 0.10 to account for small sample sizes. All means are reported ± standard error. Before constructing models to assess immune metrics, we tested if body condition varied by treatment group because physiological metrics can vary with body condition (41, 42). We estimated subject body condition by performing a linear regression of natural logarithm-transformed values of snout-vent length by body mass. We used the residuals from this regression as a proxy for body condition that accounts for differences in absolute body size (43). Because the data were normally distributed (Shapiro-Wilk test: W = 0.97, P = 0.60), we conducted an analysis of variance (ANOVA) with body condition as a response variable and treatment group as an explanatory variable.

To assess how %MKA differed by time within and among treatments, we constructed a single generalized linear mixed model with a beta error distribution for each serum+BL and microbe manipulation (e.g., %MKA against *E. coli* by frozen serum+BL) using package glmmTMB (44). Animal ID was included as a random factor. Data were converted from a 0–100 scale to a 0–1 scale to fit beta error distribution requirements. With package emmeans (45), we derived means and standard errors from each model.

To assess how total immune investment across 72 hours varied by treatment and serum+BL manipulation, we calculated two areas under the curve (AUC) for each %MKA variable (15, 46). AUCg measures investment from zero, or “the ground,” (g) whereas AUCi measures investment as an index of increase/decrease (i) from the baseline value (46). We conducted an ANOVA for each microbe and serum+BL manipulation to assess if there is a difference in immune investment across treatment groups. If tests were significant, we conducted a *post-hoc* Tukey test to assess pairwise differences.

## Results

### Body condition

Body condition did not vary by treatment group (F_24_ = 0.04, P = 0.96), so this variable was not considered in subsequent analyses.

### *E. coli* microbial killing assays

#### I. Fresh serum+BL across time within treatment group

**Figure 2:**
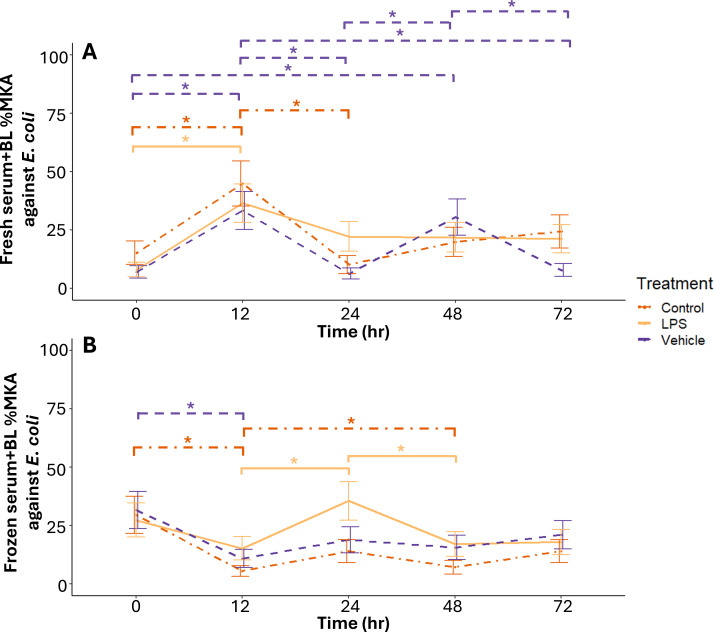
Mean ± 1 standard error %MKA against *Escherichia coli* by fresh **(A)** and frozen **(B)** serum and buffy layer samples obtained at different post exposure time points from adult male Diamond-backed Watersnakes. Significant relationships at α = 0.1 within treatment denoted with asterisks.

*Control—*Fresh serum+BL %MKA against *E. coli* from control-treated individuals was higher at 12 hours than 0 (P = 0.02) and 24 (P = 0.001) hours post-treatment (**Figure 2A**). There were no other differences (P ≥ 0.13) across time in fresh serum+BL %MKA against *E. coli* from control-treated individuals.

*Vehicle*—Fresh serum+BL %MKA against *E. coli* from vehicle-treated individuals was higher at 12 (P = 0.001) and 48 hours (P = 0.003) post-treatment than at 0 hours (**Figure 2**). Killing at 12 hours was higher than 24 (P < 0.001) and 72 hours (P = 0.003; **Figure 2A**). Fresh serum+BL %MKA was higher at 48 hours than 24 (P = 0.001) and 72 hours (P = 0.01) post-treatment (**Figure 2A**). There were no other differences across time in vehicle-treated individuals (P ≥ 0.99).

*LPS*—Fresh serum+BL %MKA against *E. coli* from LPS-treated individuals was higher at 12 hours than 0 hours post-treatment (P = 0.002; **Figure 2A**). There were no other differences across time in LPS-treated individuals (P ≥ 0.12).

#### II. Frozen serum+BL across time within treatment group

*Control*—Frozen serum+BL %MKA against *E. coli* from control-treated individuals was higher at 0 hours than 12 (P < 0.001) and 48 (P = 0.002) hours post-treatment (**Figure 2B**). There were no other differences across time in control-treated individuals (P ≥ 0.17; **Figure 2B**).

*Vehicle*—Frozen serum+BL %MKA against *E. coli* from vehicle-treated individuals was higher at 0 hours than 12 hours post-treatment (P = 0.01; **Figure 2B**). There were no other differences across time in vehicle-treated individuals (P ≥ 0.18).

*LPS*—Frozen serum+BL %MKA against *E. coli* from LPS-treated individuals was higher at 24 hours than at 12 (P = 0.05) and 48 (P = 0.10) hours post-treatment (**Figure 2B**). There were no other differences in killing *E. coli* across time (P ≥ 0.40).

#### III. Treatment comparisons

*Fresh serum+BL against E. coli*— There were no differences in fresh serum+BL %MKA against *E. coli* among treatment groups at 0 or 12 hours post-treatment (P ≥ 0.26; **Figure 2A**). At 24 hours post-treatment, fresh serum+BL %MKA against *E. coli* was higher in LPS-treated individuals than vehicle-treated individuals (P = 0.02; **Figure 2A**). There were no differences in fresh serum+BL %MKA against *E. coli* among treatment groups 48 hours post-treatment (P ≥ 0.53; **Figure 2A**). By 72 hours, however, control (P = 0. 04) and LPS-treated (P = 0.08) individuals had higher fresh serum+BL %MKA against *E. coli* than from vehicle-treated individuals (**Figure 2A**).

*Frozen serum+BL against E. coli*—There were no differences in frozen serum+BL %MKA against *E. coli* among treatment groups at 0 or 12 hours post-treatment (all P > 0.12; **Figure 2B**). At 24 hours post-treatment, frozen serum+BL %MKA against *E. coli* from LPS-treated individuals were significantly higher (P = 0.06) than control-treated individuals (**Figure 2B**). There were no other differences among treatment groups at 24, 48, or 72 hours post-treatment (P ≥ 0.20).

**Figure 3:**
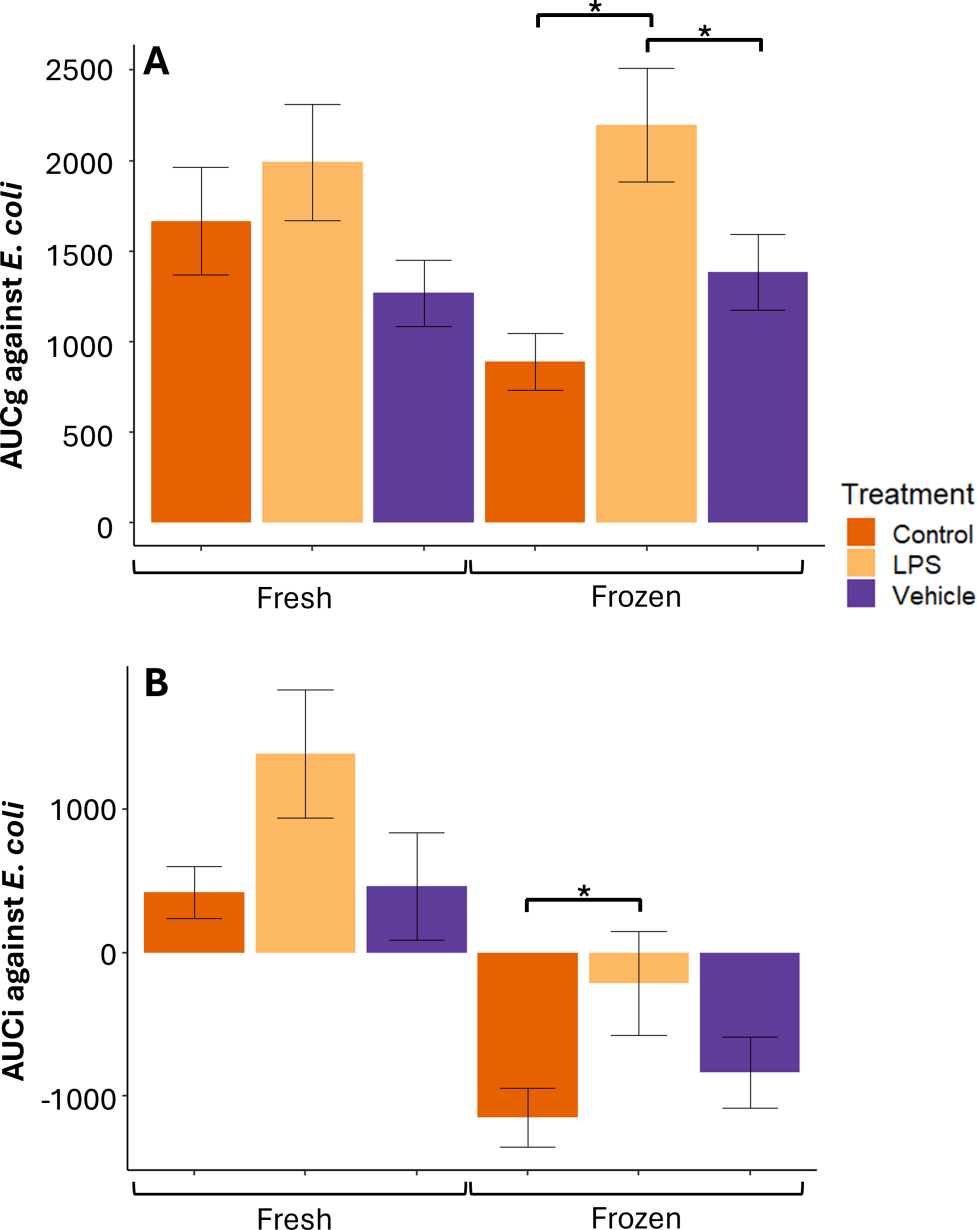
Mean ± 1 standard error *Escherichia coli* %MKA area under the curve with respect to ground (AUCg) **(A)** and area under the curve with respect to increase (AUCi) **(B)** by fresh and frozen serum and buffy layer by treatment from adult male Diamond-backed Watersnakes. Significant relationships at α = 0.1 within serum+BL manipulation denoted with asterisks.

*AUC variables*—There was no difference by treatment group in fresh serum+BL %MKA AUCg (F_25_ = 1.71, P = 0.20) or AUCi (F_25_ = 2.36, P = 0.12; **Figure 3A**) against *E. coli*. However, LPS-treated individuals had higher (F_25_ = 7.68, P = 0.003) frozen serum+BL %MKA AUCg against *E. coli* than control (P = 0.002) and vehicle-treated (P = 0.06) individuals. LPS-treated individuals also had higher (F_25_ = 2.80, P = 0.08) frozen serum+BL %MKA AUCi against *E. coli* than control-treated individuals (P = 0.07; **Figure 3B**).

### *S. aureus* microbial killing assays

#### I. Fresh serum+BL across time within treatment group

*Control*—Fresh serum+BL %MKA against *S. aureus* from control-treated individuals was higher at 0 hours than at any other sampled time points (P ≤ 0.002; **Figure 3A**). Fresh serum+BL %MKA was also higher at 48 hours than at 72 hours (P = 0.05; **Figure 3A**). There were no other differences across time (P ≥ 0.19).

*Vehicle*—Fresh serum+BL %MKA against *S. aureus* from vehicle-treated individuals was higher at 0 hours than 12, 24, 48, and 72 hours post-treatment (P < 0.001; **Figure 3A**). There were no other differences across time (P ≥ 0.64).

*LPS*—Fresh serum+BL %MKA against *S. aureus* from LPS-treated individuals was higher at 0 hours than 12 (P < 0.001), 24 (P = 0.01), 48 (P < 0.001), and 72 (P < 0.001) hours post-treatment (**Figure 3A**). Killing was also higher at 24 hours than 72 hours (P = 0.04) post-treatment (**Figure 3A**). There were no other differences in killing *S. aureus* across time (P ≥ 0.35).

#### II. Frozen serum+BL across time within treatment group

*Control*—Frozen serum+BL %MKA against *S. aureus* from control-treated individuals was higher at 12 (P = 0.01) and 72 hours (P = 0.01) than at 0 hours (**Figure 3B**). There were no other differences across time (P ≥ 0.19).

*Vehicle*—Frozen serum+BL %MKA against *S. aureus* from vehicle-treated individuals was higher at 24 hours than at 0 (P = 0.09) and 48 (P = 0.001) post-treatment (**Figure 3B**). Killing was higher at 72 hours than 48 hours post-treatment (P = 0.01; **Figure 3B**). There were no other differences in killing *S. aureus* across time (P ≥ 0.17).

*LPS*—Frozen serum+BL %MKA against *S. aureus* from LPS-treated individuals was higher at 72 hours than 0 (P = 0.001) and 48 (P = 0.07) post-treatment (**Figure 3B**). There were no other differences in killing *S. aureus* across time (P ≥ 0.11).

#### III. Treatment comparisons

**Figure 4:**
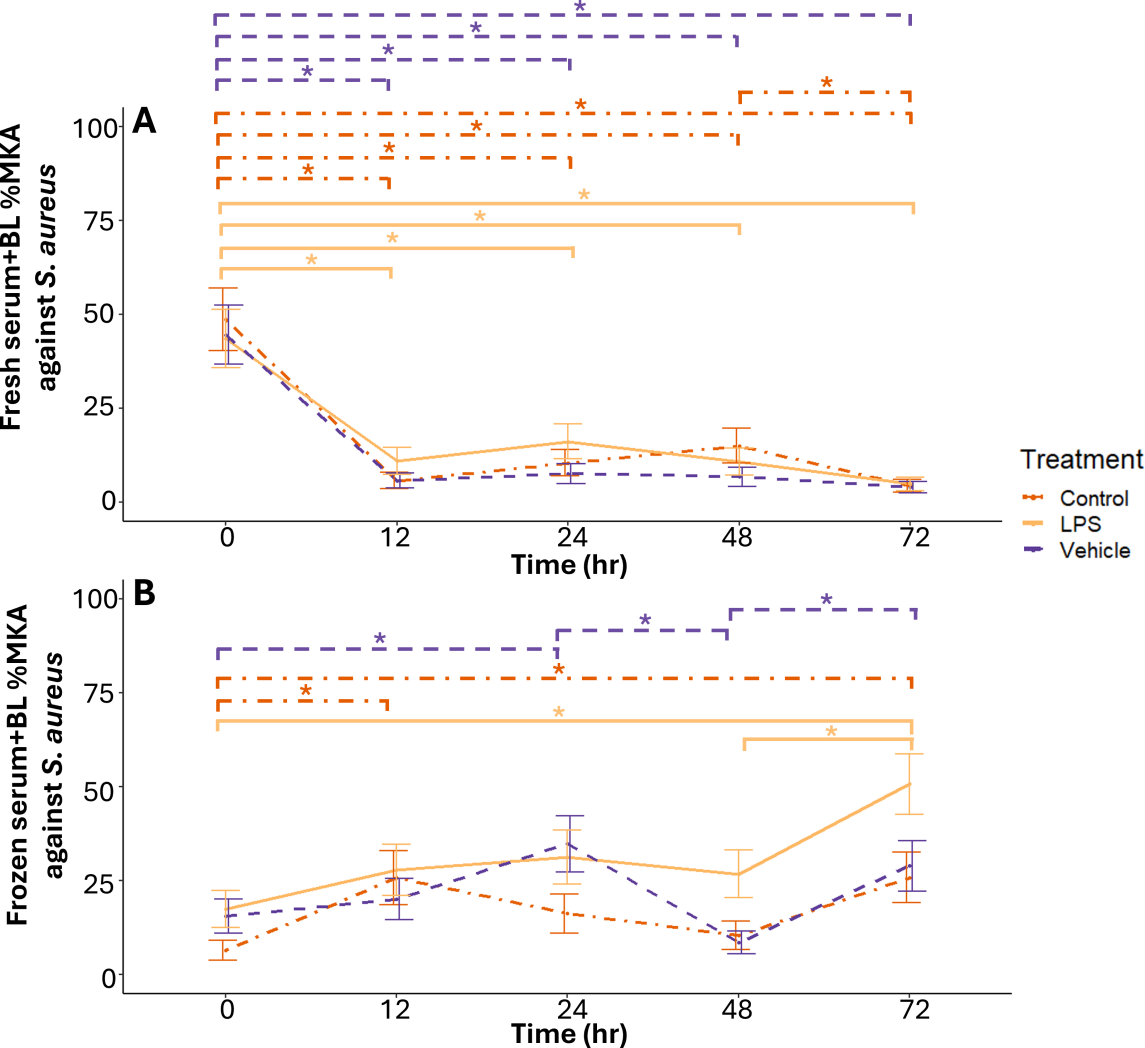
Mean ± 1 standard error %MKA against *Staphylococcus aureus* by fresh **(A)** and frozen **(B)** serum and buffy layer samples obtained at different post exposure time points from adult male Diamond-backed Watersnakes. Significant relationships at α = 0.1 within treatment denoted with asterisks.

*Fresh serum+BL against S. aureus*—There were no differences in fresh serum+BL %MKA against *S. aureus* among treatment groups at any timepoint post-treatment (P ≥ 0.19; **Figure 4A**).

*Frozen serum+BL against S. aureus*—Frozen serum+BL %MKA against *S. aureus* was higher (P = 0.09) in LPS-treated individuals prior to injection (0 hours) than control-treated individuals (**Figure 4B**). There were no other differences among treatment groups at 0-, 12-, or 24-hours post-treatment (P ≥ 0.11; **Figure 4B**). At 48 hours, frozen serum+BL %MKA against *S. aureus* was higher in LPS-treated individuals than control- (P = 0.07) and vehicle-treated (P = 0.02) individuals (**Figure 4B**). By 72 hours, frozen serum+BL %MKA against *S. aureus* was higher (P = 0.06) in LPS-treated individuals than control-treated individuals (**Figure 4B**).

**Figure 5:**
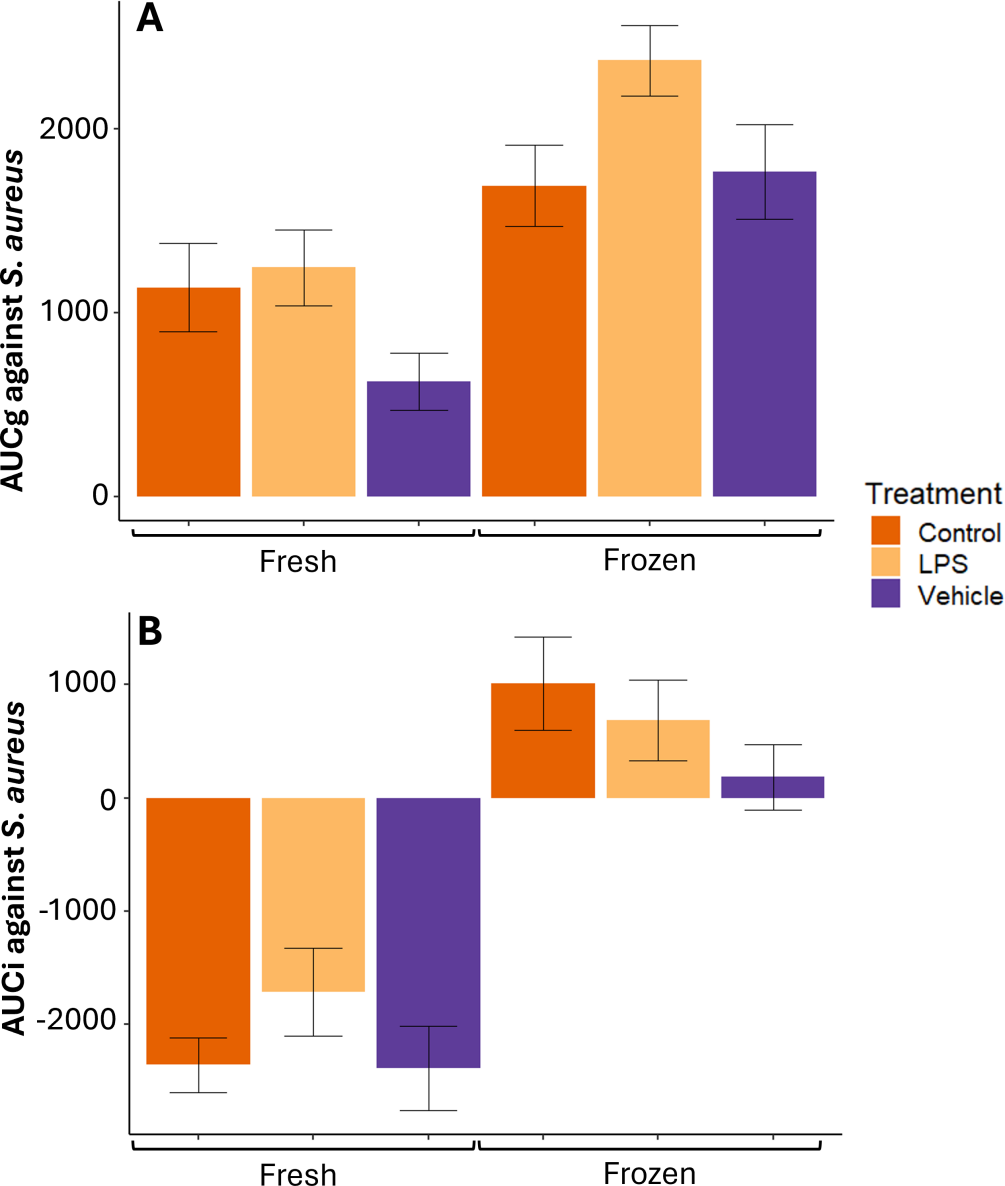
Mean ± 1 standard error *Staphylococcus aureus* %MKA area under the curve with respect to ground (AUCg) **(A)** and area under the curve with respect to increase (AUCi) **(B)** by fresh and frozen serum and buffy layer by treatment from adult male Diamond-backed Watersnakes. There were no significant relationships at α = 0.1 within serum+BL manipulation.

*AUC variables*—There was no difference by treatment group in fresh serum+BL %MKA AUCg (F_23_ = 2.45, P = 0.11) or AUCi (F_23_ = 1.27, P = 0.30) against *S. aureus* (**Figure 5**). Though the model found differences by treatment group in frozen serum+BL %MKA AUCg against *S. aureus* (F_23_ = 2.88, P = 0.08), *post-hoc* tests reveal that LPS-treated individuals were not significantly higher than control (P = 0.12) or vehicle-treated (P = 0.14; **Figure 5A**). There was no difference by treatment group in frozen serum+BL %MKA AUCi against *S. aureus* (F_23_ = 1.32, P = 0.29; **Figure 5B**).

## Discussion

**Figure 6:**
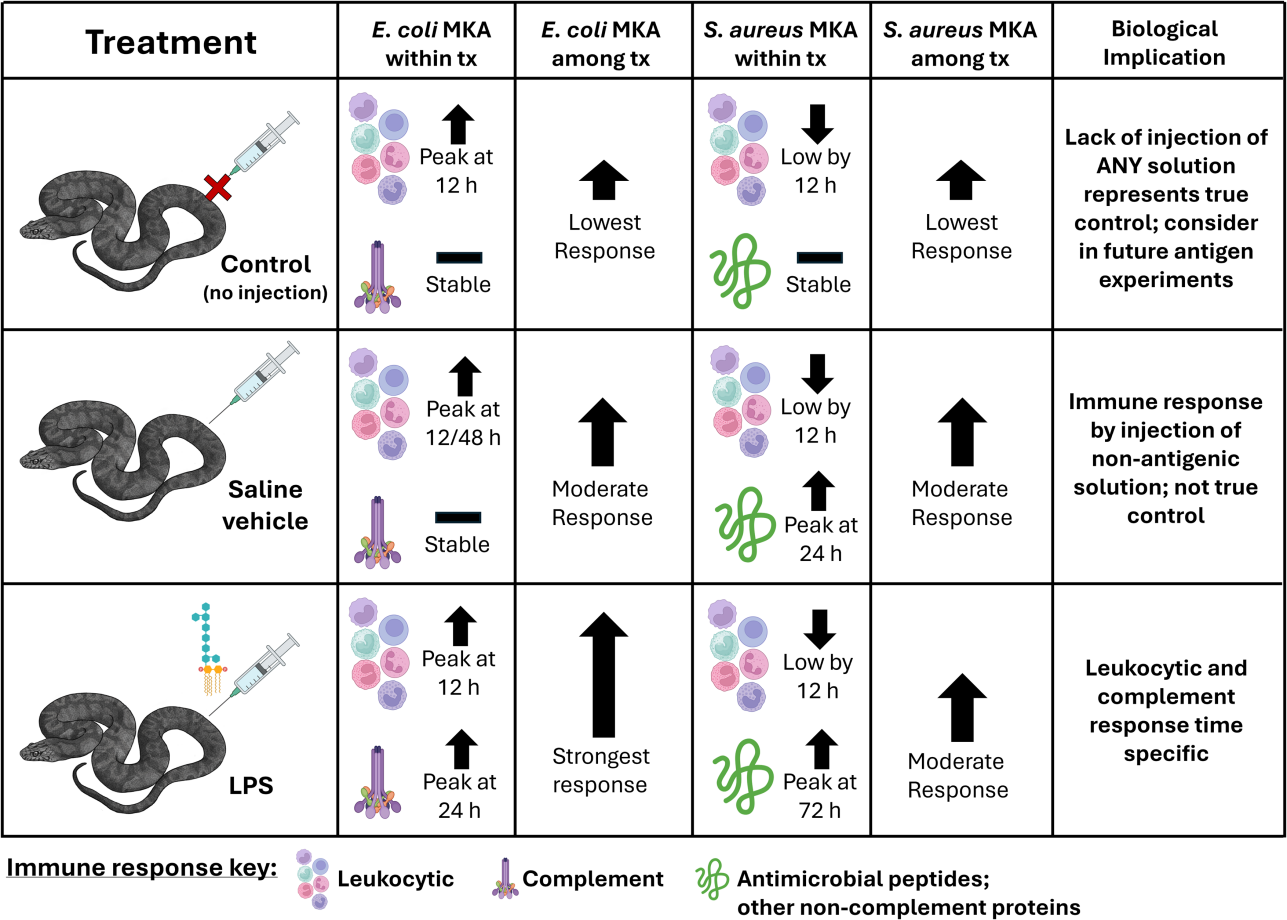
Summary of main findings in complement-dependent (*Escherichia coli* microbial killing assay; MKA) and complement-independent (*Staphylococcus aureus* MKA) responses across treatments (tx) and overall biological implication. Syringe and immune graphics from BioRender. Diamond-backed Watersnake drawing by Alyssa Hartzheim.

This study examined multiple metrics of innate immunity and how those metrics change over time in response to a simulated bacterial infection (**Figure 6**). In general, we found support for our predictions, although different immune components demonstrated different patterns. We found evidence of stronger immune responses in LPS-treated snakes specifically when the complement cascade was measured without leukocyte-dependent activity (frozen) against the gram-negative *E. coli*. Further, we observed differences in complement-dependent and complement-independent responses in that leukocyte-dependent responses to *S. aureus* decreased whereas the response increased when presented with *E. coli*. We documented an increase in protein contribution against both *E. coli* and *S. aureus* in LPS-treated snakes, however, albeit likely coming from different proteins (complement and AMPs, respectively). Finally, we found strong support that there were measurable differences in immune activity across all bactericidal treatments across the 72-hour experiment.

When examining the snake serum+BL against the complement-dependent *E. coli*, we found that the different treatments had some consistent patterns. In the leukocyte-dependent response (fresh serum+BL vs. *E. coli*), killing started at a low percentage, then increased rapidly to peak at 12 hours post-treatment. The 24 hour-post treatment saw a decrease in %MKA across all treatments, although the LPS-treated individuals maintained a higher %MKA compared to the other two groups. Although fresh serum+BL killing represents the total contribution of all immune components, we can infer contribution of leukocytes from differences between fresh and frozen samples. Patterns of cellular mobilization and redistribution in response to an immune challenge are still poorly characterized in reptiles, although studies in mammals have demonstrated clear changes in immune function of the blood (47). For example, circulating cells might migrate to the site of infection within the tissues, decreasing the immune capacity of the blood, which is often what is examined experimentally (47). Most studies examining immune cell distribution in response to LPS have demonstrated movement through blood and lymph fluid from lymphoid structures such as the spleen and lymph nodes (48). Patterns of cell movement are still unknown in reptiles, however, because most reptiles do not have permanent lymph nodes (3) and transient germinal centers have only been confirmed in some species (49). However, LPS-injected red-eared slider turtles (*Trachemys scripta elegans*) did not exhibit changes in leukocyte absolute counts from pre-injection by 72 hours (15), suggesting that redistribution may not be prolonged. The current study provides evidence that there are patterns of cellular redistribution in response to LPS repeated blood sampling, handling, probing, and/or injection.

Immune component mobilization is also evident in our current study when leukocytes are removed via freezing. Though freezing samples for one month may have also altered function of some immune proteins (50, but see 51), we observed changes in protein-based responses across time. In response to any of the treatments, circulating immune proteins had a lowered bactericidal ability against *E. coli* at 12 hours post-treatment versus the baseline. The LPS-treated individuals then had a significant increase in bactericidal ability at 24 hours post-treatment. One hypothesis for this pattern is the creation of new complement proteins formed at the liver and circulated in the blood at 24 hours. Production of complement proteins in reptiles is also poorly characterized, but mammalian complement proteins are formed both in the liver and within circulating immune cells, which can also store and secrete complement proteins such as C3 (52). Given complement’s ancient evolutionary roots (53), it is likely that reptilian immune and liver cells function in a similar manner. While mammalian complement proteins are upregulated as early as four hours post-LPS treatment, ectothermic reptiles with lower metabolisms (54) are likely to have a different time course of production. In our present study, our first point of sampling post-treatment was at 12 hours; further investigation is needed to elucidate complement production in earlier hours post-treatment.

LPS seemed to have the largest effect on the activation of the complement proteins of any of the assays we employed over the entirety of the experiment. When examining the totality of the immune response (AUC metrics; 15), we found that the LPS-treated individuals had a greater overall investment in bactericidal ability when their frozen serum+BL was challenged with *E. coli*. The high reliance on the complement cascade to clear bacterial infections has been demonstrated repeatedly in reptiles (13, 14). All three complement cascade pathways have been described in reptiles (21, 55, 56), with even closely related species preferentially using distinct pathways (55, 57). The current research adds support that LPS helps to upregulate the complement cascade in snakes, although the complement cascade is still not well-described in snakes. Because we did not partition protein-based components through the use of heat manipulation or chelation (13, 15, 23) due sample volume limitations, further work is needed to directly tease apart the relative contribution of complement and other immune proteins.

Challenging the snake serum+BL from the three different treatments to the gram-positive, complement-independent bacteria *S. aureus* provided us the ability to examine a different aspect of immunocompetence. After treatment with any of the three treatments, leukocyte-dependent immunocompetence was greatly reduced against *S. aureus* and stayed suppressed for the duration of our experiment. While the LPS-treated individuals maintained a slightly higher bactericidal ability than individuals from the other treatment groups, killing ability remained low. Pattern recognition receptors (PRRs) in reptiles are still being characterized (e.g (58). Studies have shown that there are PRRs in reptiles that detect gram-positive bacteria (e.g., toll-like receptor- 5; TLR-5 (59); and TLR-2 (60). TLR-2 is a likely candidate as a reptilian PRR for detection of the lipoproteins on *S. aureus* (61) because TLR-5 only detects flagella (62), which are not present in *S. aureus*. Most studies have not examined a wide range of taxa and/or contexts, however, so the expression and regulation of these PRRs might vary. Because PRRs are so poorly characterized in reptiles, we cannot rule out that there is PRR activation that we are not detecting. However, our data indicate that neither handling nor an LPS challenge elicit an upregulation of a gram-positive PRR, such as TLR-2, in snakes.

We detected differences within the protein immune component in response to the treatments. Because *S. aureus* is not well-cleared by complement activation (63), this assay putatively measures AMPs and NAbs when frozen serum+BL is used. We found that there was an increase in this activity over time, specifically with the LPS-treated and vehicle-treated individuals, suggesting that this increase may be due to increased AMP function based on the assumption that *S. aureus* is not well-cleared by complement (63). Antimicrobial peptides have been characterized haphazardly across reptilian taxa using various methods (20, 64, 65). While most of the AMP tested have shown high potency against gram-positive bacteria (e.g (65), some studies have demonstrated high antimicrobial peptide activity specifically against *S. aureus* (e.g., (66). We suggest that there might be patterns of AMP production that are related to handling and, potentially, to any antigenic challenge, though complement was not explicitly removed (e.g., via heat or chelating agents) from frozen samples in this study.

An experimentally elicited immune response can be critical to understanding the intricacies of the immune component contribution and time course. Reptilian immune responses are thought to be slower than endothermic counterparts because ectotherms generally have lower metabolic rates (3) and there is evidence of temperature-dependent complement function in reptiles (e.g., (67). Beyond the influence of temperature on ectothermic immune responses, there is also a key role in pace-of-life and life history influences (5). However, few studies have explicitly tested responses to antigens or pathogens over time. Studies have demonstrated changes in immune capacity as measured by thermoregulatory behavior (e.g., (32, 68, 69). bactericidal ability (15, 70), shifts in the oxidant balance (71), and changes in leukocyte ratios (72). Additionally, LPS injection has been shown to influence factors such as metabolic rate (38, 73).

We have demonstrated the importance of including a vehicle and control group in manipulative studies. We found increased immune responses in the group that was injected with saline (vehicle) compared with the group that was not injected and only probed with needle cap. Given that the act of needle injection causes damage to tissue, there are likely certain damage-associated molecular patterns (DAMPs) activated (74). Although we are unaware of any DAMPs definitively identified in reptiles, they have been identified in plants, insects, fish, and mammals (74–77). Furthermore, although the saline solution was sterile, there was an introduction of additional fluid and salts, which can alter immune responses, typically through suppression of inflammatory responses (78, 79). Observed immune responses to vehicle solutions may also vary due to solute composition or concentration. Although our study cannot explicitly assess the difference in effect of injection action (puncture) or injection solution (saline), it emphasizes the need to include at least vehicle and control groups during experimental design. Optimally, future projects should consider the use of a control (handling only with needle cap probe), needle-only (puncture with needle; no solution), and a vehicle group (injection with saline) to fully elucidate responses to needle-based injury, DAMP activation, and antigen treatment. We documented altered immune responses consistent with handling effects across all treatments, specifically in the enhanced complement-dependent activity against *E. coli* and suppressed leukocyte-dependent activity against *S. aureus* (80). While the nuances of which components actually elicited different responses across the three treatments (e.g., DAMPs, handling, antigen) cannot be definitively measured here, differences in the LPS-treated individuals were apparent when comparing to both the vehicle and control.

The current study increases our understanding of reptilian immunity and how varied the different innate immune components are. Our findings underscore the need for an increased use of more immune assays to provide a clearer picture of the overall immune response. We corroborated other studies that have demonstrated a high reliance of protein-based immunity in reptiles, particularly complement activation (e.g 13–15, 23).

## Data Availability

The raw data supporting the conclusions of this article will be made available by the authors, without undue reservation.
